# Angioedema, an unusual reaction to hair dye

**DOI:** 10.11604/pamj.2018.30.103.12061

**Published:** 2018-06-06

**Authors:** Reginald Mzudumile Ngwanya, Zandile Spengane, Nonhlanhla Khumalo

**Affiliations:** 1 Division of Dermatology, Groote Schuur Hospital, University of Cape Town, Observatory Cape Town, South Africa

**Keywords:** Angioedema, henna, facial swelling, paraphenyldiamine, patch test

## Abstract

Angioedema is a type-1 hypersensitivity reaction that can be life threatening. It affects the skin airways and the gastrointestinal tract. Henna is a green powder used to dye skin and nails. We report a 29 year old patient who presented with angioedema Her patch test was positive to paraphenynlenediamine. She was discharged after successful treatment with intravenous steroids and later topical steroids.

## Introduction

Paraphenynlenediamine (PPD) a potent skin sensitizer is a cause of allergic contact dermatitis, a type-IV hypersensitivity reaction. It is a constituent of hair dyes and is also added to red henna thereby creating black henna. Skin reactions to PPD include angioedema urticarial and anaphylactic reactions. There are systemic manifestations of PPD poisoning such as acute kidney injury, rhabdomyolysis and toxic myocarditis. Angioedema, a type-1 hypersensitivity reaction, is characterised by swelling of the skin and subcutaneous tissue. In addition to skin it affects respiratory and gastrointestinal systems and presents with laryngeal swelling that can be life threatening. It is important to recognise the condition early so not to give inappropriate treatment.

## Patient and observation

**Figure 1:**
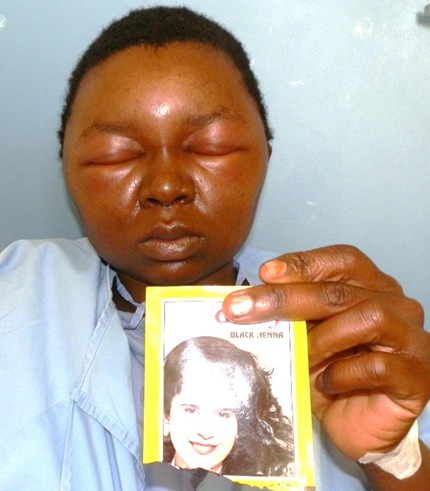
Clinical photograph showing swollen face

**Figure 2:**
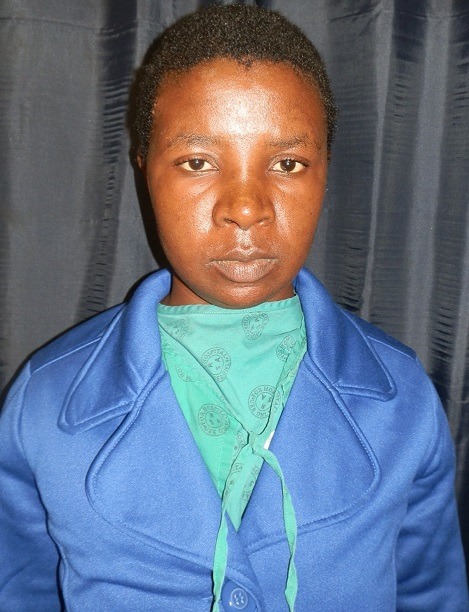
Clinical photograph 5 days later

**Figure 3:**
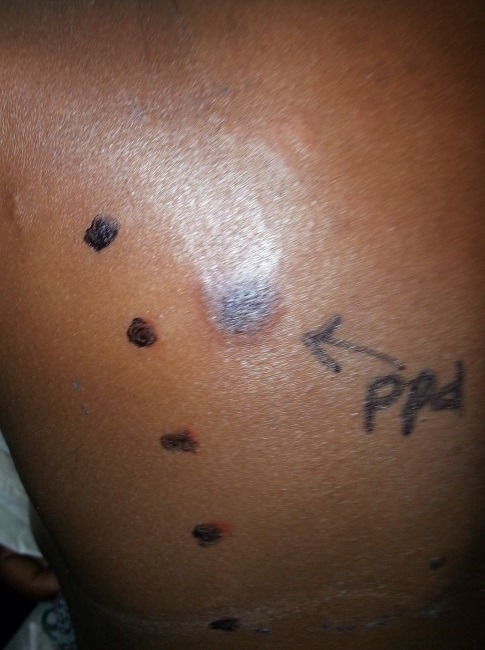
Standard patch test showing a positive reaction to PPD

A 29 year old lady presented to the Dermatology Department at Groote Schuur Hospital with a 2 day history of severe swelling of her face, marked periorbital oedema which developed overnight. She had used henna hair dye containing paraphenynlenediamine (PPD) the previous day. There was no history of previous sensitization with the use of hair dye or tattoos using black henna. Patient was initially treated at a pharmacy and given Amoxicillin for a presumed diagnosis of cellulitis. She was then later taken to a day hospital in the early morning on Day 2 of symptoms where she received hydrocortisone 200mg intravenously and Phenergan 25mg intramuscularly both as stat doses. This made no improvement and the patient was referred to a tertiary hospital. On arrival at Groote Schuur hospital she was given repeat hydrocortisone 100mg intravenously and Phenergan 25mg intramuscularly. She had severe facial oedema with marked periorbital oedema (Figure 1). There was no swelling of the tongue. She had scale crust on her scalp. Skin punch biopsy was unremarkable. The patient was admitted into our dermatology ward and put on prednisone 40mg orally daily, Vitamin D weekly, calcium tablets and chlorpheniramine maleate 4mg daily. Topically she received cetomacrogol cream to de-crust her scalp. Once de-crusted, clobetasol cream was applied. She also applied clobetasol cream to her face for 4 days which was weaned down to methylprednisolone on day 5 and weaned down to 1% hydrocortisone ointment on day seven. The patient made a full recovery by day 5 and discharged on Cetomacrogol and fluocinolone gel for scalp and 1% hydrocortisone ointment for the face (Figure 2). A standard patch test that was subsequently done showed positive patch test to PPD (Figure 3).

## Discussion

Reactions to hair dyes are common and range from mild to severe and life threatening. They include contact dermatitis, angioedema and anaphylaxis. These are caused by PPD contained in hair dyes. The complications of PPD include airway obstruction. PPD poisoning causes rhabdomyolysis, methaemoglobin, toxic myocarditis and acute renal failure [1]. An angioedema-like allergic contact dermatitis related to black henna has been reported before. This was a 23 year old man who had a mild contact dermatitis to black henna tattoo with prior sensitization [2]. There was no history of prior sensitization in our patient. Henna is a green powder obtained from a plant [3]. It stains the skin reddish brown hence it is called red henna. It appears to be safe though contact allergic reactions had been described rarely. No natural black henna exists and black henna is a combination of henna with PPD. PPD is a known sensitizer and PPD reactions can result in death. Sensitization had occurred even after black henna tattoos [4,5]. High sensitization rates have been found in certain occupations such as hairdressers and workers in wood, glass and chemical industries.

There is cross reactions with allergens such as azo dyes found in leather, textiles, fur and rubber products. It is important to dilute PPD when doing a patch test to avoid sensitization. There had been a linear trend in the increased sensitization to PPD in patch tests over the years from 2% to 7% [6]. Because of the dangers associated with PPD, it is banned in most European countries such as Germany, France and Sweden and has been labelled an occupational health hazard [1]. It is recommended that lower concentrations between 0.5%-1 percent PPD should be used in patch tests for those who had previously used henna tattoos and in children and must be left for 24 hours [7]. Most people are unaware of the differences between red and black henna. They may also be unaware of the dangers of PPD in black henna. It is important for strict labelling of henna as to the presence of PPD.

## Conclusion

It is important for the physicians to be aware of the differential diagnosis of a red swollen face that it is not cellulitis only and angioedema-like reactions should be included as these would require a different approach to the management. The learning points in this case highlight the fact that the differential diagnosis of facial swelling must also include contact dermatitis to hair products. Accurate diagnosis is needed for correct and prompt treatment and labelling of black henna should state the presence of PPD and the dangers linked with its use.

## Competing interests

The authors declare no competing interests.
